# The Information Consistency Between Full- and Improved Dual-Polarimetric Mode SAR for Multiscenario Oil Spill Detection

**DOI:** 10.3390/s25175551

**Published:** 2025-09-05

**Authors:** Guannan Li, Gaohuan Lv, Tong Wang, Xiang Wang, Fen Zhao

**Affiliations:** 1 Ulsan Ship and Ocean College, Ludong University, Yantai 264025, China; lvgh@ldu.edu.cn; 2 College of Computing and Data Science, Nanyang Technological University, Singapore 639978, Singapore; tongwang@ntu.edu.sg; 3 Ocean Remote Sensing Division, National Marine Environmental Monitoring Center, Dalian 116026, China; xwang@nmemc.org.cn; 4 School of Resources and Environmental Engineering, Ludong University, Yantai 264025, China; zhaof@ldu.edu.cn

**Keywords:** marine oil spills, dual-polarimetric synthetic aperture radar, comparative analysis, information consistency

## Abstract

Detecting marine oil spills is vital for protecting the marine environment, ensuring maritime traffic safety, supporting marine development, and enabling effective emergency response. The dual-polarimetric (DP) synthetic aperture radar (SAR) system represents an evolution from single to full polarization (FP), which has become an essential tool for oil spill detection with the growing availability of open-source and shared datasets. Recent research has focused on enhancing DP information structures to better exploit this data. This study introduces improved DP models’ structure with modified the scattering vector coefficients to ensure consistency with the corresponding components of the FP system, enabling comprehensive comparison and analysis with traditional DP structure, includes theoretical and quantitative evaluations of simulated data from FP system, as well as validation using real DP scenarios. The results showed the following: (1) The polarimetric entropy *H_L_* obtained through the improved DP scattering matrix *C_L_* can achieve higher information consistency results closely aligns with FP system and better performance, compared to the typical two DP scattering structures. (2) For multiple polarimetric features from DP scattering matrix (both traditional feature and combination feature), the improved DP scattering matrix *C_L_* can be used for oil spill extraction effectively with prominent results. (3) For oil spill extraction, the polarimetric features-based *C_L_* tend to have relatively high contribution, especially the *H_A* feature combination, leading to substantial gains in improved classification performance. This approach not only enriches the structural information of the DP system under VV–VH mode but also improves oil spill identification by integrating multi-structured DP features. Furthermore, it offers a practical alternative when FP data are unavailable.

## 1. Introduction

With the growth of marine resource development and the shipping industry, marine oil spill accidents have become increasingly frequent [1,2]. These incidents—caused by ship collisions [3], natural seabed seepage [4], and platform releases [5]—have drawn widespread public concern due to their notable effects on the marine ecological environment, maritime traffic, aquaculture, oil exploration, and related sectors [6,7,8,9]. Therefore, the rapid and effective identification of oil spills is critical for marine environmental protection, maritime safety, and oil field development.

The polarimetric synthetic aperture radar (SAR) system exhibits all-day and all-weather microwave imaging capabilities, especially under adverse weather conditions such as cloud cover and fog [10,11,12]. It can transmit and receive electromagnetic pulses in two orthogonal polarization channels and record holographic data, including intensity and coherent phase information, from different polarization modes [6,10]. Its detection capability and information richness surpass those of single-polarimetric systems. Many studies have been conducted on oil spill detection based on full polarimetric (FP) systems, covering various types of features [4], and natural oil seep [10], different types of sensors conditions [11], different types of oil slicks [13], etc. However, due to the increased pulse repetition frequency required by FP systems, challenges arise in balancing data acquisition efficiency, transmission and processing requirements, system power consumption, antenna technology, and image swath width [14,15].

The dual-polarimetric (DP) system bridges the transition from single- to full-polarimetric SAR systems. As the DP system captures only partial polarization information, which lacks information of a certain polarimetric channel combination compared to the FP system. Therefore, in terms of target information richness, scattering mechanism description, and physical significance, the DP system has certain limitations. Moreover, the DP systems with different channel combinations also have differences in scattering mechanism descriptions, it poses challenges for interpreting target mechanisms, extracting polarimetric features, and effectively identifying targets [16,17]. Although DP SAR offers a low-complexity, cost-effective solution with a broad imaging coverage and efficient data processing—achieved by sacrificing complete polarization information—many studies continue to explore its potential based on this tradeoff. On one hand, researchers aim to extract feature parameters suited for DP systems by introducing and extending full-polarimetric structures [13,18,19,20]. On the other hand, efforts have been made to improve the scattering vector constructs of DP systems to enhance their information richness—making them more comparable to FP systems [16,17,20]. The dual co-polarization system has emerged as a viable alternative to fully polarimetric systems and is widely applied in earth observation. For instance, in polarimetric information extraction, Skrunes et al. (2013) proposed modified polarimetric parameters derived solely from the co-polarized complex scattering structure, demonstrating good ability in distinguishing between biogenic and mineral oils [13].

Furthermore, the importance of the vertical transmit–vertical receive and vertical transmit–horizontal receive (VV–VH) system in environmental monitoring is growing rapidly. Researchers are actively investigating the VV–VH model to deepen understanding of target recognition potential within DP systems and to enhance their information richness, aiming to achieve information consistency comparable to FP systems [16,20,21,22]. With the availability of open-source data from DP satellites like the Sentinel series, applications have expanded to land classification, biomass estimation, crop monitoring, oil pollution detection, and earthquake-related scattering analysis [16,23,24,25,26,27,28,29,30,31,32,33,34,35]. The scattering structure, originally proposed by Cloude for FP systems [36], has been adapted to DP and widely applied in detecting ground objects such as oil spills, vegetation, and crops, etc. [17]. In structural improvement, Ji and Wu further developed an improved DP scattering vector structure, successfully applied to soil state identification [20]. However, theoretical analysis and comparisons among different polarization scattering vector structures remain limited. Building on Ji and Wu’s improved *C_J_* matrices [20], Liang [16] proposed the *C_L_* DP scattering structure, which more closely aligns with the coefficient configuration of the FP matrix. This was supported by theoretical derivation and analysis, demonstrating its closer resemblance to FP system characteristics [16].

Conventional studies typically compare different polarimetric modes, such as full-polarimetric versus a specific DP mode, often focusing on a single scenario. Moreover, improved versions of these modes generally rely on aggregated multitarget scenarios, lacking targeted research on marine-specific information. However, with the recent expansion of polarimetric modes, few studies have comprehensively compared multiple oil spill scenarios across different polarimetric modes. Therefore, this study focuses on a comprehensive analysis of multisource marine oil spill scenarios under various polarization modes. It conducts comparative analysis based on FP data converted to different simulated DP modes, as well as on real DP data. The goal is to reveal how the performance of different DP modes in the recognition of typical oil spill information and assess their consistency with FP information, supporting effective alternative approaches when FP data are unavailable. The primary contributions of this study are as follows:

- A detailed comparison of information representation and abundance among different improved DP modes; analysis of consistency and differences between DP—and FP modes; and examination of similarity representations across DP modes.
- The introduction of FP features into improved DP structural modes to evaluate the feature extraction and adaptation of homologous parameters across multiple polarimetric modes with multisource oil spill data.
- A comprehensive analysis of the commonly polarimetric features from different DP structures is conducted for statistical analyses of oil spill information and the separability, contrast, classification ability, and classification contribution.

The remainder of the paper is organized as follows: Section 2 describes the collection of multisource oil spill scenario data. Section 3 outlines the theoretical foundations of improved DP systems under various scattering structures and analyzes their relationships with FP systems. Section 4 presents qualitative and quantitative analyses of oil spill detection performance across different polarimetric modes and scenarios, including a comparison of recognition capabilities in real DP systems. Section 5 discusses key points related to this work, and Section 6 concludes the paper.

## 2. Dataset Description

**Figure 1:**
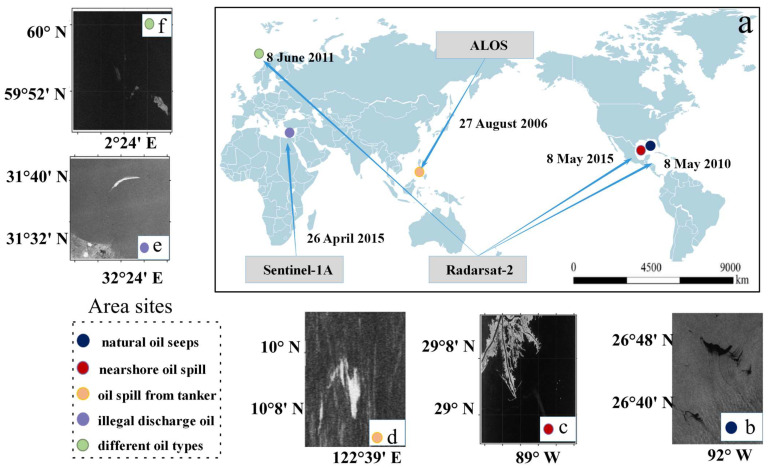
Overview of area sites. (**a**) Locations of the five oil spill sites; (**b**) Gulf of Mexico (natural oil seeps); (**c**) Gulf of Mexico (nearshore oil spill); (**d**) Philippines (oil spill from a tanker); (**e**) Suez Canal (illegal discharge); and (**f**) North Sea, Norway (artificial experiments with different oil types).

In order to ensure a rigorous and representative comparative analysis of oil spills, we carefully considered key factors such as typical oil spill scenarios, geographic sea areas, and commonly used radar bands. Specifically, we utilized C-band single-look complex RADARSAT-2 FP-mode images, L-band ALOS PALSAR FP-mode images, and C-band Sentinel-1A VV-VH DP mode data. The selected polarimetric SAR data represent five typical oil spill scenarios: natural oil seeps in the Gulf of Mexico, manufactured oil on water experiment involving different oil types in the North Sea of Norway, accidental nearshore oil spills, a heavy oil spill incident off the coast of the Philippines, and real oil spill scenarios data from Sentinel-1A DP mode. These datasets have been used in various studies, including analyses of relative thickness differences, characteristics of different oil types, and oil spill accidents in nearshore areas. The causes of the oil spills include natural seepage, controlled experimental spills, and real accident events. Scenarios 1–4 are FP datasets used to simulate DP mode under different spill conditions, while Scenario 5 consists of real DP data used to verify the effectiveness of the proposed method. This study aims to use these datasets to conduct a comprehensive analysis across different oil spill formation mechanisms, radar bands, and representative spill scenarios in various sea areas. Table 1 and Figure 1 provide detailed information about all SAR images used in this study under these diverse oil spill scenarios.

In this study, for each oil spill scenario, we randomly selected 10,000 sample points from specific target areas in the five oil spill images for statistical analysis and comparative evaluation, using the “Create Random Points” tool in ArcGIS 10.2. The analyses included mean, standard deviation, contrast measure, and overlap degree. For the Sentinel-1A image, 50% of the samples were utilized for classification training and modeling, and the remaining 50% for accuracy verification.

## 3. Method

**Figure 2:**
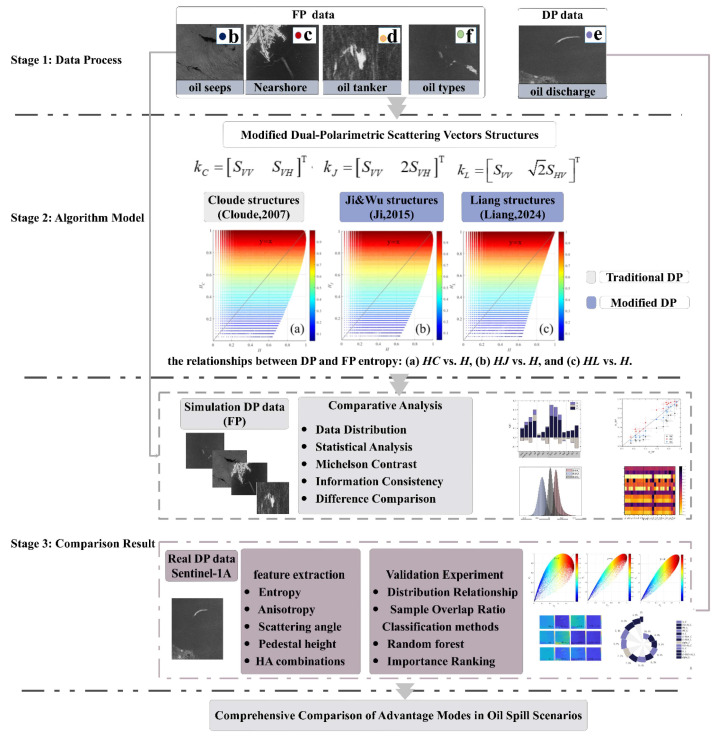
Flow charts outlining the proposed method [16,17,20].

Figure 2 shows the flow chart of the proposed method. The method mainly consists of three steps: in the first step, the preprocessing of different oil spill scenarios data has been carried out, and the corresponding FP covariance matrices have been obtained for future comparison. In the second step, the improved scattering structure is introduced and compared with the traditional one, including the structural composition and theoretical relationship. In the third step, qualitative and quantitative comparisons are conducted in both simulated and real DP data, including the consistency relationship with FP data and the advantages of different DP mode under multi-source oil spill scenarios.

### 3.1. The Structure from FP Data

The FP SAR system provides four linear combinations of transmit and receive polarization channels and has been widely applied in oil spill detection and interpretation across various oil spill scenarios. The full scattering matrix (*S*) of a pure (single) target, and its corresponding Lexicographic-basis scattering vector (*k_L_*) (a three-dimensional vector) under a FP SAR system, are defined as follows [10]:

(1) $$S=\left[\begin{array}{cc} S_{HH} & S_{HV}\\ S_{VH} & S_{VV} \end{array}\right]\Rightarrow K_{L}={\left[\begin{array}{ccc} S_{HH} & \sqrt{2}S_{VH} & S_{VV} \end{array}\right]}^{T}$$

Here, the elements *S_ij_* (where *i*, *j* ∈ {H,V}) denotes scattering coefficients, with the former and latter subscript indicating the received polarization and transmitted polarization, respectively. *H* and *V* stand for horizontal and vertical polarizations, respectively [10]. Under the reciprocity assumption (i.e., *S_HV_* = *S_VH_*), the three-dimensional Lexicographic-basis scattering vector *k_L_* encapsulates the polarimetric backscattering information and its associated physical properties of the scattering target [10].

The average covariance matrix (*C*_3_), constructed as the outer product of *k_L_* and its conjugate transpose $k_{L}^{*T}$, is given by

(2) $$C_{3}=\langle k_{L}k_{L}^{*T}\rangle =\langle \left[\begin{array}{ccc} C_{11} & C_{12} & C_{13}\\ C_{12}^{*} & C_{22} & C_{23}\\ C_{13}^{*} & C_{23}^{*} & C_{33} \end{array}\right]\rangle \;=\left[\begin{array}{ccc} \langle {\left|S_{HH}\right|}^{2}\rangle & \sqrt{2}\langle S_{HH}S_{HV}^{*}\rangle & \langle S_{HH}S_{VV}^{*}\rangle \\ \sqrt{2}\langle S_{HV}S_{HH}^{*}\rangle & 2\langle {\left|S_{HV}\right|}^{2}\rangle & \sqrt{2}\langle S_{HV}S_{VV}^{*}\rangle \\ \langle S_{VV}S_{HH}^{*}\rangle & \sqrt{2}\langle S_{VV}S_{HV}^{*}\rangle & \langle {\left|S_{VV}\right|}^{2}\rangle \end{array}\right]$$

### 3.2. Structure from Improved DP Data

In a DP system, the scattering vector structure is initially derived from the Cloude model, which was developed for a FP system [17]. The corresponding covariance matrix can be directly constructed using the Lexicographic basis, similar to that of a FP system:

(3) $$k_{C}={\left[\begin{array}{cc} S_{VV} & S_{VH} \end{array}\right]}^{T}$$

(4) $$\begin{array}{l} {\langle C_{VV-VH}\rangle }_{C}=\frac{1}{L}\sum_{i=1}^{L}k_{C}k_{C}^{H}\\ =\left[\begin{array}{cc} \langle {\left|S_{VV}\right|}^{2}\rangle & \langle S_{VV}S_{VH}^{*}\rangle \\ \langle S_{VH}S_{VV}^{*}\rangle & \langle {\left|S_{VH}\right|}^{2}\rangle \end{array}\right] \end{array}$$

For the improved structures proposed by Ji &Wu [20] the covariance matrix with the corresponding scattering vectors under the Lexicographic basis are expressed as

(5) $$k_{J}={\left[\begin{array}{cc} S_{VV} & 2S_{VH} \end{array}\right]}^{T}$$

(6) $$\begin{array}{l} {\langle C_{VV-VH}\rangle }_{J}=\frac{1}{L}\sum_{i=1}^{L}k_{J}k_{J}^{H}\\ =\left[\begin{array}{cc} \langle {\left|S_{VV}\right|}^{2}\rangle & 2\langle S_{VV}S_{VH}^{*}\rangle \\ 2\langle S_{VH}S_{VV}^{*}\rangle & 4\langle {\left|S_{VH}\right|}^{2}\rangle \end{array}\right] \end{array}$$

Liang et al. [16] inspired by the research of Ji and Wu, further improved the scattering vector coefficients with the scattering matrix, are expressed as

(7) $$k_{L}={\left[\begin{array}{cc} S_{VV} & \sqrt{2}S_{VH} \end{array}\right]}^{T}$$

(8) $$\begin{array}{l} {\langle C_{VV-VH}\rangle }_{L}=\frac{1}{L}\sum_{i=1}^{L}k_{L}k_{L}^{H}\\ =\left[\begin{array}{cc} \langle {\left|S_{VV}\right|}^{2}\rangle & \sqrt{2}\langle S_{VV}S_{VH}^{*}\rangle \\ \sqrt{2}\langle S_{VH}S_{VV}^{*}\rangle & 2\langle {\left|S_{VH}\right|}^{2}\rangle \end{array}\right] \end{array}$$

In addition, the covariance matrices for the conventional DP mode (*C_C_*) [17], Ji & Wu’s model (*C_J_*) [20], and Liang’s model (*C_L_*) [16]—along with their correlations to the elements of the FP matrix—are summarized in Table 2.

The factor $\sqrt{2}$ in Equation (7) ensures power consistency between Equation (8) and the VV–VH channel components of FP in Equation (9), which aligns with the actual polarimetric scattering characteristics of the observed target. This also indicates that Liang’s model has clear physical significance, as it matches the structural coefficients and channel components of the FP covariance matrix. In practical applications, it yields physical quantities closer to FP results, thereby improving the information consistency and richness of the DP system.

(9) $${\left[C_{L}\right]}_{\left(2,2\right)}={\left[C\right]}_{\left(2,2\right)}=\frac{1}{2}*{\left[C_{J}\right]}_{\left(2,2\right)}=2{\left[C_{C}\right]}_{\left(2,2\right)}$$

(10) $${\left[C_{L}\right]}_{\left(1,2\right)}={\left[C\right]}_{\left(2,3\right)}=\frac{1}{\sqrt{2}}*{\left[C_{J}\right]}_{\left(1,2\right)}=\sqrt{2}{\left[C_{C}\right]}_{\left(2,2\right)}$$

(11) $${\left[C_{L}\right]}_{\left(1,1\right)}={\left[C\right]}_{\left(3,3\right)}={\left[C_{J}\right]}_{\left(1,1\right)}={\left[C_{C}\right]}_{\left(1,1\right)}$$

### 3.3. Comparative Analysis and Feature Extraction

From the structural analysis and comparison of the scattering vector and the corresponding covariance matrix, it can be observed that the DP modes with different scattering vector structures, the coefficient of *C_L_* is consistent with the corresponding element in the FP covariance matrix. Therefore, further exploration and discussion are necessary through comparative analysis of multisource oil spill scene information.

For the DP systems under the three modes, the maximum eigenvalue $\lambda_{\left(C,J,L\right)}$ and the corresponding maximum pseudo-probability $P_{\left(C,J,L\right)}$ can be derived as follows [16]:

(12) $$\lambda_{\left(C,J,L\right)}=\frac{1}{2}*\left({\left[C_{\left(C,J,L\right)}\right]}_{\left(1,1\right)}+{\left[C_{\left(C,J,L\right)}\right]}_{\left(2,2\right)}+\sqrt{{\left({\left[C_{\left(C,J,L\right)}\right]}_{\left(1,1\right)}-{\left[C_{\left(C,J,L\right)}\right]}_{\left(2,2\right)}\right)}^{2}+4*{\left({\left[C_{\left(C,J,L\right)}\right]}_{\left(1,2\right)}\right)}^{2}}\right)$$

(13) $$P_{\left(C,J,L\right)}=\frac{1}{2}+\frac{\sqrt{{\left({\left[C_{\left(C,J,L\right)}\right]}_{\left(1,1\right)}-{\left[C_{\left(C,J,L\right)}\right]}_{\left(2,2\right)}\right)}^{2}+4*{\left({\left[C_{\left(C,J,L\right)}\right]}_{\left(1,2\right)}\right)}^{2}}}{2\left({\left[C_{\left(C,J,L\right)}\right]}_{\left(1,1\right)}+{\left[C_{\left(C,J,L\right)}\right]}_{\left(2,2\right)}\right)}$$

Polarimetric entropy represents the degree of statistical disorder associated with each of the different scattering types in the ensemble. The polarimetric entropy of the DP system is derived from the FP system [17]:

(14) $$H_{DP}=-\sum_{i=1}^{2}p_{DPi}\log_{2}\left(p_{DPi}\right)=-p_{DP}\log_{2}\left(p_{DP}\right)-\left(1-p_{DP}\right)\log_{2}\left(1-p_{DP}\right)$$

Although the DP mode data lacks some polarimetric information, and the coefficients of the corresponding elements in the covariance matrices of the three DP modes are different, their covariance matrices also preserve the feature as a principal submatrix of the FP covariance matrix. Therefore, the DP entropy and the FP entropy have similar physical significances and performance on the corresponding targets. For example, water bodies corresponding to a single scattering mechanism show lower scattering complexity with lower entropy values, while mountainous areas with obvious volume scattering show higher complexity with higher entropy values. The extraction formulas with entropy for the FP and the DP modes are slightly different due to the difference in the number of eigenvalues. Analyzing and comparing the consistency of the three DP entropies with the FP entropy can deepen the interpretation of the scattering mechanism and the characteristics of the targets.

For the comparative analysis of different DP modes, this paper conducts a comparison of various polarimetric features based on the simulation DP model from FP data and the real oil spill scenarios DP data (Sentinel-1A). It conducts a comprehensive assessment of aspects such as the data distribution between FP-DP data, contrast, information consistency, data overlap degree, and classification contribution.

## 4. Results and Analysis

### 4.1. Theoretical Relationships Between FP and DP Entropy Under Different Structures

**Figure 3:**
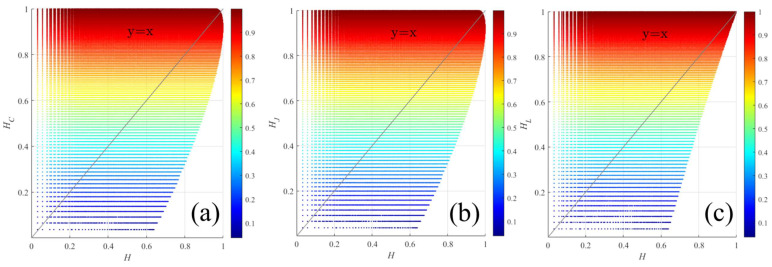
Simulation results of the relationships between DP and FP entropy, respectively: (**a**) *HC* vs. *H*, (**b**) *HJ* vs. *H*, and (**c**) *HL* vs. *H*. The gray line in each figure is the angle bisector of X and Y axes.

According to (12) and (13), the *P_L_*, *P_C_*, and *P_J_* under three DP modes can be directly obtained. Based on the corresponding relationship of (9)–(11), the common sub-components in the three DP models include *r_C_* = $\langle {\left|S_{VV}\right|}^{2}\rangle$/$2\langle {\left|S_{VH}\right|}^{2}\rangle$ corresponding to $C_{33}$/$C_{22}$ in the FP matrix, and *r_X_* = ${\left|\langle S_{VV}S_{VH}^{*}\rangle \right|}^{2}$/$2{\langle {\left|S_{VH}\right|}^{2}\rangle }^{2}$ corresponding to ${\left|C_{23}\right|}^{2}$/$C_{22}^{2}$ in the FP matrix. In brief, the relationships among *P_L_*, *P_C_*, and *P_J_*, as well as among *H_L_*, *H_C_*, and *H_J_*, can be transformed into one another and subsequently calculated. This allows for the direct comparison of the polarimetric entropy obtained from the FP system with that from different DP structure systems [16]. The simulation results are shown in Figure 3. Overall, the uncertainty relationship between *H_L_* and *H* decreases as *H* increases. The relationship between *H_C_*, *H_J_* and *H* is similar to the characteristic distribution of *H_L_*, but its distribution range becomes larger with the increase in *H* compared to *H_L_* [16].

### 4.2. Information Consistency Comparison Between FP and DP Entropy

To verify these theoretical relationships using real oil spill scene data, and to analyze the information consistency between FP and DP, this study used FP data from different sea areas and various oil spill accident scenarios to simulate corresponding DP structures. The dataset covers multiple frequency bands and different oil spill scenarios. In addition, real VV–VH mode oil spill data is adopted to construct different DP structures for comparison. This approach follows general methods used in related research [4,12,13,16].

Based on the above, and disregarding the missing HH channel components in the three modes, the structure of Liang’s *C_L_* is consistent with that of the FP system, while the coefficient of CC is less than 1/$\sqrt{2}$ times, and the coefficient of *C_J_* is greater than $\sqrt{2}$ times. This indicates that the polarimetric information of the target in the CL structure is closer to that in the FP system [9]. The coefficients of *C_C_* and *C_J_* are, respectively, lower and higher than the target information in the FP system. However, since *C_L_* contains a higher proportion of the cross-polarization channel than *C_C_*, the influence of noise introduced by the cross-polarization channel on distinguishing target information remains uncertain when identifying ground objects. This section verifies the information consistency between DP entropy and FP entropy through qualitative and quantitative analyses.

#### 4.2.1. Comparative Analysis and Verification Based on Simulated DP Oil Spill Data

**Figure 4:**
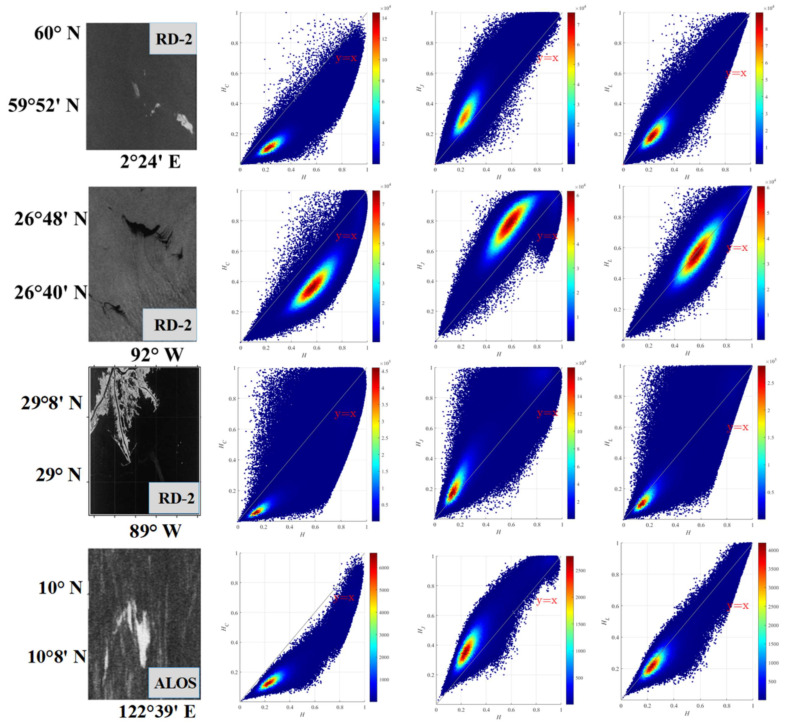
Comparison of entropy results between DP entropies and FP entropy H under different oil spill scenarios: the first column is an original visualization of different oil spill scenarios, the second column are *HC* vs. *H*, the third column are *HJ* vs. *H*, the fourth column are *HL* vs. *H*.

Using FP entropy (*H*) as the reference, the scatter plots comparing FP and DP entropies across different structures are shown in Figure 4. Overall, the distribution of data across various oil spill scenarios generally aligns with the expected theoretical ranges and boundaries. Furthermore, the distribution patterns remain relatively consistent across different oil spill scenarios. Within the DP system, the information consistency between *H_L_* and *H* is particularly strong, with most data points clustering near the line y = x and displaying symmetrical distribution on either side of this line. In contrast, *H_C_* and *H_J_* tend to be, respectively, higher and lower than *H*. Moreover, scenarios 1, 2, and 4 consist mainly of seawater and oil slick data, showing distributions towards one side of the main diagonal. Scenario 3 includes land, seawater, and oil spills, resulting in a more complex ground feature mix; accordingly, its data distribution follows a pattern consistent with the theoretical range [16].

**Figure 5:**
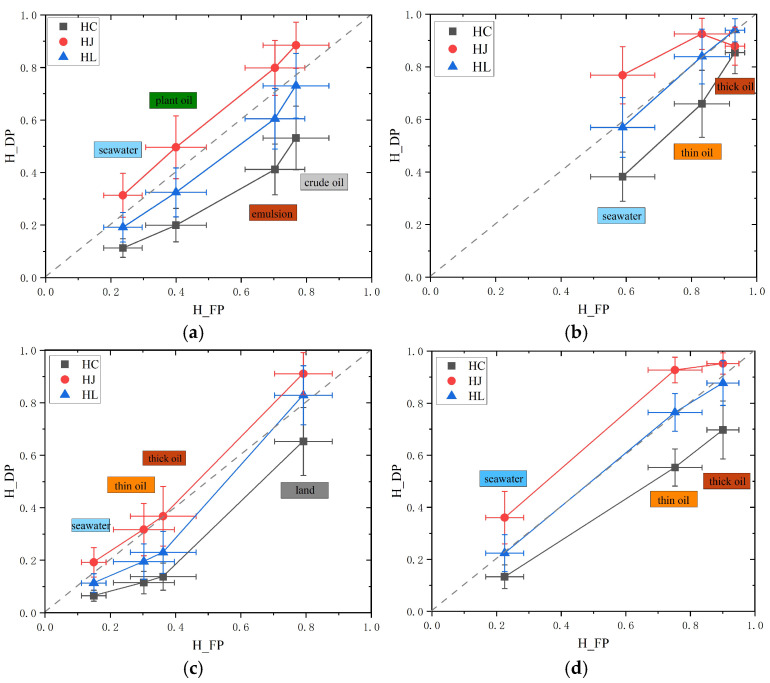
Relationships between FP and DP entropies in selected sample areas across different oil spill scenarios. (**a**) Different oil types; (**b**) natural crude oil seeps; (**c**) nearshore oil spill; (**d**) heavy oil spill from a tanker.

Furthermore, the corresponding visualizing relationships between DP entropies and FP entropy H on the sample areas from different oil spill scenarios, are shown in Figure 5, which, respectively, compare DP entropies with FP entropy in different oil spill scenarios samples with error bars provided. For various oil spill scenarios, comparing the results of *H* with those of DP entropy reveals that *H_J_* is significantly higher than *H*, while *H_C_* is significantly lower than *H. H_L_* and *H* generally exhibit a relatively consistent distribution, although in some cases, *H_L_* shows slightly lower fluctuations even when they are closely aligned. By comparing the differences among the DP entropy values, it is generally observed that *H_J_* is higher than *H_L_* and *H_C_*, while *H_L_* tends to be closer in value to *H_C_*. However, in the thick oil regions of the natural oil seep area, the overall *H_J_* image appears as a high-entropy region with relatively large noise, leading to a noticeable deviation in the mean value for that area. Additionally, in the nearshore oil spill accident scenario, the *H_J_* value in the oil slick area is closer to the FP *H* than *H_L_*. These observations are further supported by the subsequent comparison of consistency-based quantitative indicators. Considering that the dataset includes multisource scenarios across different bands, oil spill regions, and causes of oil spills, *H_L_* can reasonably be regarded as an effective descriptor of the randomness in target polarimetric scattering. It also demonstrates better information consistency with the FP system, which is consistent with the previous research results [16].

#### 4.2.2. Difference Comparison Experiment Based on Different Entropies

**Figure 6:**
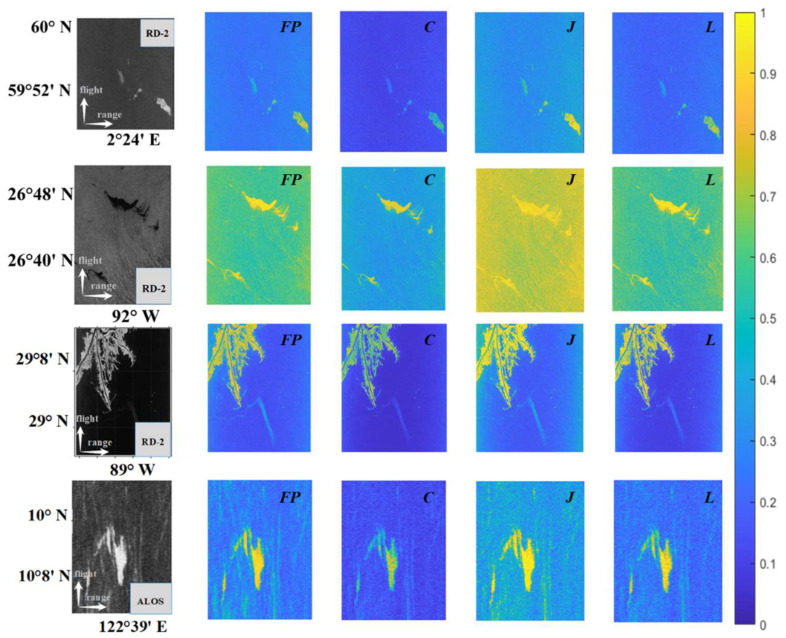
Visualization results of polarimetric entropy *H* between full- and dual-polarimetric systems with different scattering structures under various oil spill scenarios. The rows under various oil spill scenarios: different oil types, oil seep, nearshore oil spill, and oil spill from a tanker.

For oil spill monitoring and identification, the most potential features are determined by evaluating quantitative indicators such as information consistency, intraregional clustering degree, and inter-regional differences. Figure 6 present the *H* results of different polarimetric modes across various oil spill scenarios captured by Radarsat-2 and ALOS. In the matrix of entropy maps, each rows corresponds, in sequence, to natural oil seep (with relative thickness differences), artificial oil spill experiments (with different oil types), nearshore oil spill accidents, and L-band oil spill accidents (e.g., a tanker spill), and each column from left to right corresponds *H*, *H_C_*, *H_J_*, and *H_L_*, respectively. From the visualization results, *H_L_* is observed to be consistent with the FP system, in terms of overall data distribution and single target category. Overall, the value of *H_J_* is higher than the other two DP entropies. This is attributed to its highest proportion of cross-polarization components, leading to higher randomness across seawater, land, and oil spill regions—particularly in the natural oil seep scenario. In contrast, *H_C_* exhibits lower randomness due to its matrix having lower element coefficients for cross-polarization channels compared to the other two structures. Thus, the visualization results indicate that *H_L_* aligns more closely with FP information overall. These observations are quantitatively supported by the results shown in Table 3.

**Figure 7:**
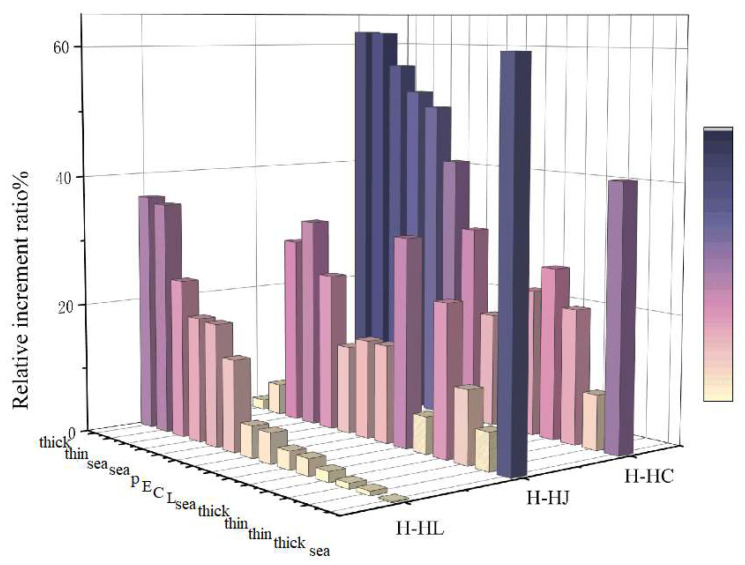
Relative increment ratio result of polarimetric entropy *H* between FP and DP systems with different scattering structures under various oil spill scenarios.

**Figure 8:**
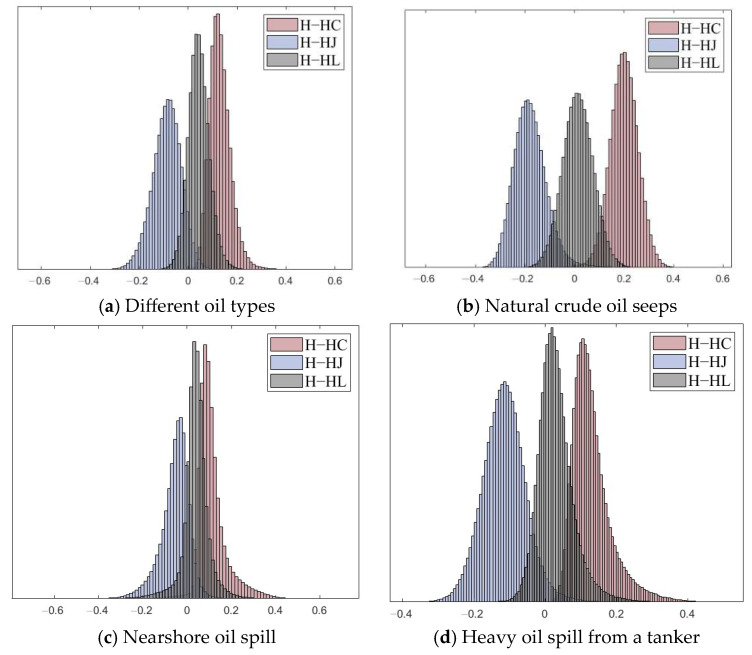
Histogram distribution of the differences in polarimetric entropy *H* between full- and dual-polarimetric modes using different structural configurations under various oil spill scenarios: (**a**) difference oil types, (**b**) oil seep, (**c**) nearshore oil spill, and (**d**) oil spill from a tanker.

The numerical comparison showed that the entropy values of both the seawater and oil spill area in Table 3, we compute the mean and standard deviation of *H*, *H_C_*, *H_J_*, and *H_L_*. The values with a blue background indicate FP entropy, while boldface highlights the result that is closest to full polarization among the DP structures *C*, *J*, and *L*. By comparing the FP entropy and DP entropy values under different DP systems, *H_L_* generally shows the closest match to the FP entropy, with the difference being minimal in most cases. *H_C_* is typically lower than the FP entropy, whereas *H_J_* is usually higher. However, *H_J_* aligns more closely with FP data in certain complex scenarios, such as scenarios 1 (Norway experiment) and 3 (Nearshore oil spill). The oil spill situation is more complex. When emulsified oil and oil slicks are thoroughly mixed with seawater, the data tends to resemble the FP results more closely. Moreover, in most target scenarios, the *H_J_* data exhibits greater divergence compared to *H_C_* and *H_L_*. Between the latter two, *H_L_* and *H_C_* data are relatively similar. This conclusion is also reflected in the corresponding Figure 7 and Figure 8. The differences between FP entropy and DP entropies vary. The distribution results show that *H_L_* and the FP entropy *H* share consistent characteristics. Their difference follows a normal distribution centered around zero. The best consistency appears in the natural oil seep scene, while other scenarios show slightly positive differences, indicating that *H_L_* is slightly higher than *H*. In contrast, *H_J_* and *H_C_* differ significantly from *H*. Since *H_J_* is significantly higher, the distribution of differences tends to be negative. Conversely, *H_C_* is generally lower than *H*, resulting in a positively skewed difference distribution. The mean and standard deviation effectively reflect the deviations between the entropy values of different DP modes and the FP entropy. Among them, *H_L_* demonstrates better consistency with the FP across various oil spill scenarios compared with *H_C_* and *H_J_*.

Furthermore, the differences between the DP entropies and FP entropy H were quantitatively assessed across different oil spill scenarios. The Michelson Contrast (MC) measure [37], originally developed to evaluate contrast differences between targets within the same image, has been widely used in image classification and target recognition [13,37]. In the context of polarimetric SAR oil spill monitoring, the MC measure compares two datasets, *i* and *j*—where a larger MC value indicates a greater difference between them, and a smaller value indicates a lesser difference. In this study, the MC measure is applied to evaluate the degree of difference between the entropy values of various DP structures and FP entropy image. The formula is expressed as follows:

(15) $$MC=\frac{F_{FP}-F_{DP}}{F_{FP}+F_{DP}}$$

For the same target sample area, *F_FP_* denotes the full-polarimetric feature parameter and *F_DP_* represents the same parameter in different DP modes. Equation (16) omits the absolute value operation to preserve the original data magnitude for direct comparison. Consequently, the sign of the outcome indicates direction: a positive value means the FP entropy is greater than the DP entropy, while a negative value indicates the opposite.

**Figure 9:**
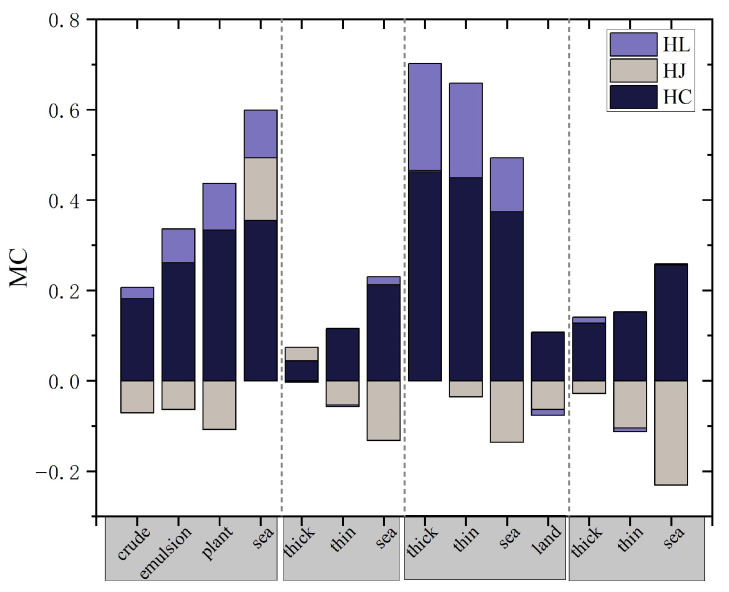
Comparison of the MC measure for polarimetric entropy *H* between FP and DP systems with different structures under various oil spill scenarios.

The results are presented in Table 4 and Figure 9. Across different oil spill scenarios, *H_C_* values are consistently lower than FP entropy, and their MC measurements are all positive. For most targets, *H_J_* values are higher than FP entropy, resulting in mostly negative MC measurements. *H_L_* values are generally consistent with FP entropy in most cases, with only slight fluctuations, which can also be observed from the visual results in Figure 6. Since MC measures the difference between DP entropies and FP entropy *H*. *H_L_* produces the lowest MC values for most targets among the three DP modes, indicating the smallest deviation from FP. This aligns with the results in Figure 8, which also show *H_L_* achieving the best consistency with FP. However, in certain cases—such as emulsions (Norway experiment) and oil slick targets (Nearshore oil spill)—*H_J_* values are closer to FP than *H_L_*. This may be due to thinner oil slicks in these cases, which are fully mixed with seawater and exhibit more complex scattering mechanisms, resulting in higher *H_J_* values relative to the other two modes and closer to FP entropy under such conditions. Figure 9 visually illustrates the MC measurement differences, showing that in most conditions the difference ratio between *H_L_* and FP is the smallest.

### 4.3. Comparison of Different Polarimetric Feature Parameters

Building on the comparison of polarimetric entropy, we introduced FP parameters into various DP modes to obtain corresponding parameter sets for a comprehensive comparison. We selected the overlap degree measure between target samples as the evaluation index to quantify the confusion between target samples across different features. It is defined as

(16) $$OR=\frac{m_{i\&j}}{m_{i}}$$

where *m_i&j_* represents the sample statistics number of overlapping parts of the two types of targets *i* and *j*, and mi represents the total sample number of corresponding various targets under the condition of sample overlap. In this study, all samples were selected with the same total number for quantitative analysis.

**Figure 10:**
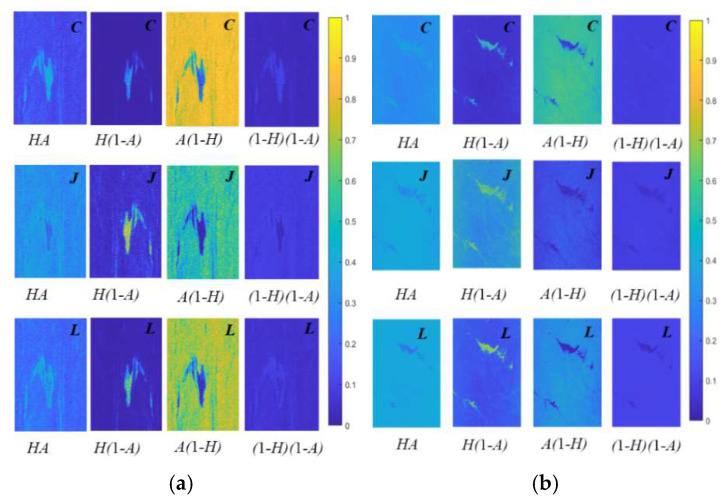

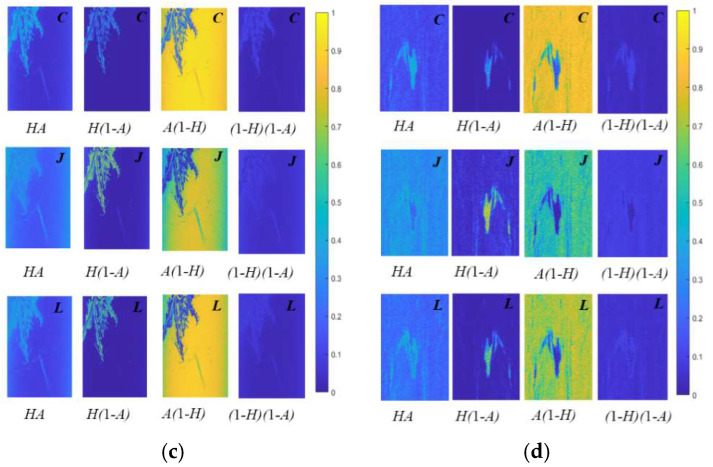
Comparison results of the *H*_*A* combination feature in different oil spill scenarios with different DP modes: the rows are Cloude’s, Wu’s and Liang’s model, respectively. (**a**) Different oil types; (**b**) natural crude oil seeps; (**c**) nearshore oil spill; (**d**) heavy oil spill from a tanker.

**Figure 11:**
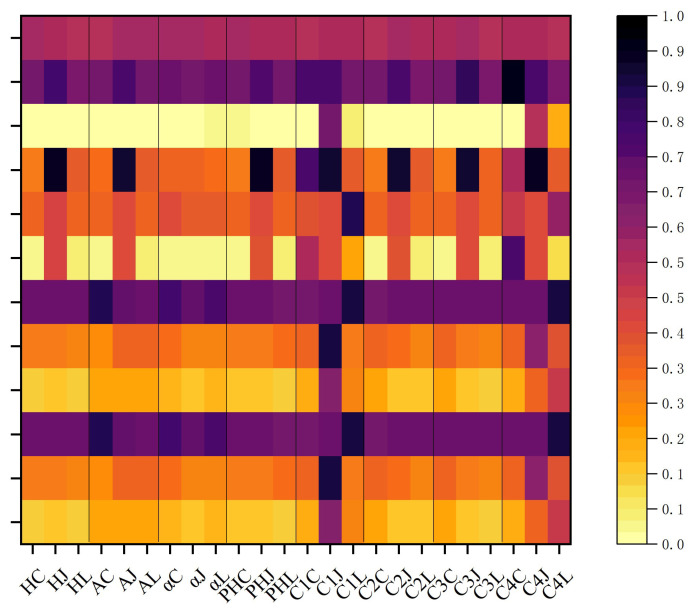
Heat maps showing the overlap degree of feature parameters under different DP modes.

As presented in Table 5 and Figure 10 and Figure 11, for common polarimetric feature parameters, Liang’s mode generally performs better or is close to the optimal result, particularly in terms of polarimetric entropy and pedestal height. It also achieves strong results in distinguishing oil slicks from seawater, as well as mineral oil from plant oil. This suggests that Liang’s mode has strong potential for identifying and differentiating oil slicks from look-alikes. It is noteworthy that Ji and Wu’s mode performs well in polarimetric scattering angle (α) results, making it a viable choice for future feature analyses. For the four combination features of entropy (*H*) and anisotropy (*A*), the three DP modes demonstrate different oil spill identification capabilities. Overall, the combination feature under Liang’s mode shows better, and in some cases superior, performance compared with Cloude’s mode, achieving the optimal result. However, target contrast is slightly lower under Ji and Wu’s mode. In detail, Combination 2 and Combination 3 correspond to *H*(1 − *A*) and A(1 − *H*), respectively. Liang’s mode performs best under most conditions, followed by Cloude’s mode. Under the *H* × *A* combination, results for Liang’s and Cloude’s modes are similar, both performing well in natural seep oil and nearshore oil spill scenarios. The (1 − *H*)(1 − *A*) combination exhibits relatively weak discrimination across oil spill scenarios, with significantly higher overlap ratios between targets under all three modes compared with other feature parameters. The comprehensive overlap degree heat map clearly shows that Liang’s and Cloude’s modes outperform Ji and Wu’s mode in various oil spill scenarios, with Liang’s mode achieving the overall most optimal results.

### 4.4. Experiment with Sentinel-1 DP Oil Spill Scene Data

**Figure 12:**
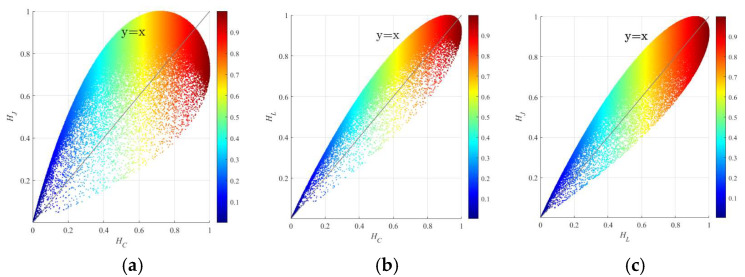
Comparison results of the relationships between different H with Sentinel-1A data. (**a**) *H_C_* and *H_J_*; (**b**) *H_C_* and *H_L_*; (**c**) *H_L_* and *H_J_*.

To further explore and analyze the oil spill identification capabilities of different DP structure, this study selects Sentinel-1A oil spill data to perform feature extraction under various DP scattering structures. The study focuses on the northern entrance of the Suez Canal as the research area and uses Sentinel-1 images for comparison experiments. Based on the theory presented in Section 3, three DP structures were constructed from the Sentinel-1A data. The relationships among the DP entropies are shown in Figure 12, and their distribution and corresponding boundaries align with the theoretical simulation results [16]. The distributions of *H_L_* and *H_J_*, as well as *H_L_* and *H_C_*, are relatively close, whereas *H_C_* and *H_J_* exhibit greater divergence, indicating notable differences between them. This is also reflected in the significant differences in the element coefficients of their polarimetric matrices. According to Equations (9)–(11), the relationships and coefficient ratios among the elements in the covariance matrices for the three modes are shown. The coefficients of Liang’s matrices fall between those of Cloude’s and Ji & Wu’s. Consequently, *H_L_*’s distribution range is similar to both Cloude’s and Ji & Wu’s, as illustrated in Figure 12b,c. However, the coefficients of Cloude’s and Ji & Wu’s differ substantially, resulting in more scattered distribution ranges in Figure 12a, as explained in Section 3 [16].

**Figure 13:**
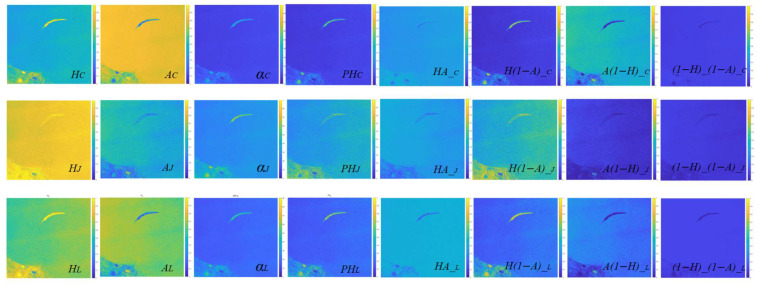
Visualization of multi-polarimetric features under different DP modes with Sentinel-1A data. The rows are Cloude’s, Wu’s and Liang’s model, respectively. The columns correspond to entropy *H*, anisotropy *A*, scattering angle α, pedestal height *PH*, and the four mathematical combinations of *H* and *A*, namely H*A, H*(1−*A*), A*(1−*H*), and (1−*H*)*(1−A).

Consistent with the simulation comparison results, covariance matrices of different DP structures were constructed from the Sentinel-1A oil spill data. Commonly used polarimetric features and corresponding feature combination sets were obtained, and the visualization results alongside quantitative separation degree indicators were comprehensively compared. The results are shown in Figure 13 and Table 6. In relatively thick oil slick areas, thin oil slick areas near the edges, and seawater, optimal results were mainly achieved with polarimetric features from Liang’s covariance matrix *C_L_* and Cloude’s covariance matrix *C_C_*, particularly for polarimetric entropy, anisotropy, and the *HA* combination. Overall, features extracted from *C_L_* typically yield the lowest overlap degree, followed by those from *C_C_*, with Ji & Wu’s features performing best in only a few cases. Moreover, within the *HA* combinations, *H* (1 − *A*) and *A* (1 − *H*) consistently deliver better results. These findings indicate that feature parameters derived from the *C_L_* structure produce optimal or near-optimal results across different target contrasts.

### 4.5. Classification Experiment Based on Different Polarimetric Features with Sentinel-1A Data

**Figure 14:**
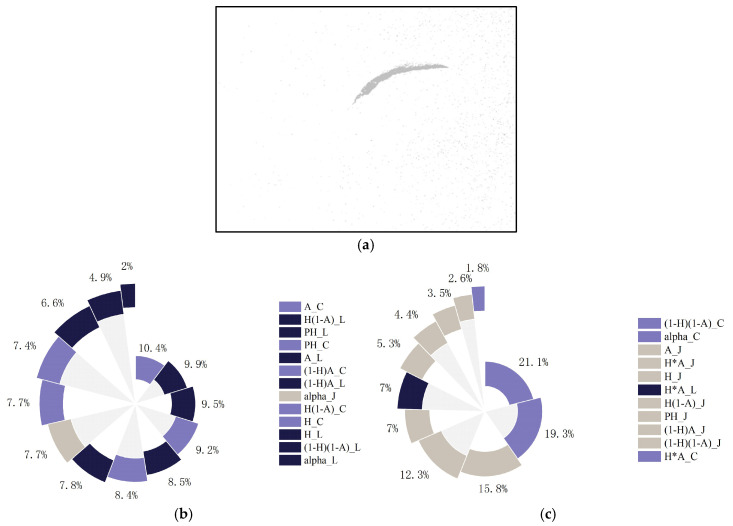
Results of the random forest classifier and importance ranking of polarimetric features. (**a**) Oil spill detection results; (**b**) high contribution group; (**c**) low contribution group.

Oil spill detection results and the variable importance rankings of scenarios with different DP modes using Sentinel-1A data are shown in Figure 13 and Table 7, respectively. Overall, these visualization results and accuracy indicators demonstrate effective classification and extraction of mineral oil and seawater. However, there are still some misclassifications in the results, such as image edge noise, the edge and outermost of the oil slick area. On the one hand, it is likely due to increased noise that alters scattering randomness. Due to the large incident angle with Sentinel-1 data (results in the small radar cross-section), the measurement deviation may be more pronounced for the ocean surface, which was also observed by Liang et al. [16]. Additionally, the edge of oil slick is subject to the emulsification, weathering and diffusion, which is classified as seawater. However, the edges of the image over the sea were misclassified as oil slicks, likely due to increased noise that alters scattering randomness. The feature importance rankings corresponding to the classification results are shown in Figure 14. The 24 features covering the *C*, *J*, and *L* structures display a stepwise distribution, divided into a first echelon and a second echelon. Almost half of the features in the first echelon derive from the *C_L_* structure, while only one feature from the *C_L_* structure appears in the second echelon. Moreover, the *C_L_* features hold a clear numerical advantage among the most important features. In summary, the DP features of the *C_L_* structure have similar importance to those of the *C_C_* structure and generally demonstrate better overall performance.

## 5. Discussion

### 5.1. Consistency of Information Between Different DP Structures and FP Structures

This study extended the DP model, previously applied to land-use classification, to typical marine targets such as marine oil spills. It explored the level of consistency between information from DP and FP systems, as well as the performance of dual polarization in identifying marine oil spills. The consistency between DP and FP systems was examined from two perspectives: theoretical comparison of scattering structures and the performance of polarimetric feature parameters. First, the correspondence between different DP modes and the FP system was evaluated based on theoretical structure. The coefficients of the elements in the *C_L_* matrix correspond to those in the FP covariance matrix. Thus, *C_L_* is closer to the FP system than the other two DP structures under the same oil spill scenarios. Second, polarimetric entropy was used as a reference to compare the three DP structures with FP. Both qualitative and quantitative analyses were performed, based on visualization results, statistical distributions, relative increment ratios, relative difference histograms, and MC measures across different target samples and oil spill scenarios. The results indicated that *H_L_* generally outperforms the other two DP structures in terms of data distribution, overall data dispersion status, and performance across oil spill scenarios, showing the best consistency with FP. However, *H_L_* was slightly lower than *H_J_* in some complex oil–water mixture sample areas. Therefore, different targets can be identified and detected to achieve optimized feature utilization by combining multi-source feature fusion. Although *H_L_* demonstrated strong consistency with FP, it exhibited marginally lower entropy in multisource oil spill scenarios, likely due to the HH channel’s influence in enhancing target randomness. Skrunes and Hoogeboom [13,38,39] concluded that: *The backscatter decreases faster in HH than in VV, which can explain the larger difference between the two channels*. Therefore, *H_L_*—with the same coefficient—has slightly less entropy overall than full-polarimetric *H*. By contrast, *H_C_* and *H_J_* show higher randomness (i.e., higher entropy values) due to the greater contribution of cross-polarization channel coefficients. Among the DP structures, *H_L_* lies between *H_C_* and *H_J_*, consistent with the coefficient analysis from the theoretical evaluation.

### 5.2. Performance of Oil Spill Polarimetric Features with Different DP Scattering Structures

Considering that a single polarimetric entropy parameter is insufficient to analyze the ability of different DP structures to identify oil spill characteristics. In this study, experiments were conducted to evaluate the capability of various DP structures to recognize oil spill information. Eight polarimetric features commonly used in oil spill monitoring were selected, including both single-polarization and combination features [40]. We found that features extracted by the covariance matrix under the *C_L_* structure achieved optimal or near-optimal results across different oil spill scenarios, performing particularly well with the *H_A* combination features. In contrast, features extracted by the covariance matrix under the *C_J_* structure showed diminished effectiveness in identifying oil spills, due to an increased influence of cross-polarization components. Additionally, single indicators such as the target overlap degree had limited ability to fully describe the characteristics of oil spill targets. The simulation data analysis was complemented by classification experiments using Sentinel-1A oil spill data to enrich the evaluation of the classification performance of DP structures. The experimental results aligned well with the quantitative evaluations from the simulation data. While the features based on covariance matrix *C_C_* and *C_L_* contributed most to the classification results, *C_L_* performed nearly as well as *C_C_*. Among the high-contribution gradient indicators, *C_L_* accounted for the majority, followed by *C_C_*. However, within the low-contribution indicators, only one feature belonged to the *C_L_* structure. These results indicate that features from the *C_L_* structure make substantial contributions to classification performance.

## 6. Conclusions

To address the limitations of DP systems in extracting only partial polarization information for oil spill monitoring—due to their inherent structural constraints—we introduced an improved method for extracting polarimetric features based on different scattering vector structures. This method aims to enhance information consistency between DP and FP systems. The performance of different scattering structures was evaluated using eight commonly used polarimetric features across four typical oil spill scenarios, employing both qualitative (visualization results and histograms) and quantitative (MC measure, overlap degree, classification accuracy, and variable importance ranking) assessment methods. The experiments validated the effectiveness of the proposed method and demonstrated its advantages in extracting oil spill information using simulated DP data derived from FP data under multi-source conditions, as well as actual DP oil spill data. The main contributions of this study are summarized as follows:

1. For DP systems with different scattering structures and for FP systems under multi-source oil spill scenarios, when polarimetric scattering entropy *H* is used as a reference, the *H_L_* under improved structure shows higher information consistency with the FP data compared with the *H_C_* and *H_J_* structures. Across various oil spill scenarios, the clustering behavior of *H_L_* in target samples aligns more closely with fully polarimetric *H*, exhibiting similar means and variances. Whether in qualitative comparisons (visualization and distribution ranges) or quantitative analyses (statistical indicators and contrast results), *H_L_* generally shows the greatest consistency with FP data.
2. In comparing the effectiveness of different DP modes for oil spill detection—whether based on simulated or actual DP data—the polarimetric features extracted using the *C_L_* covariance matrix consistently produced optimal or near-optimal results compared with those obtained using *C_C_* and *C_J_* covariance matrices. Among the homology-defined polarimetric parameter sets from the three structures, the features extracted using the *C_L_* covariance matrix accounted for the highest proportion of high-contribution features.

Last but not least, the combined use of polarimetric feature parameters across different scattering structures in DP systems shows promise not only for land classification but also for marine target detection, particularly in oil spill identification. However, variations in oil spill causes, affected sea areas, sea conditions, and environmental factors result in different detection characteristics. Leveraging multiple scattering structures can effectively expand the information capacity of DP systems, enhance recognition ability, and play a vital role in rapid oil spill detection, environmental restoration, and post-disaster emergency response. Therefore, the optimal mode combination should be selected according to the specific operational context. Future work will further validate the advantages of the proposed improved structure by examining its universality and scenario-specific performance. This will include testing additional polarization modes such as HH–VH, a wider range of oil spill conditions, and various platforms such as TerraSAR-X, as well as different acquisition settings (e.g., incidence angle and instrument noise) and oil spill scenarios (e.g., oil type, accident origin, region, and formation mechanism). In addition, in-depth research will focus on polarimetric feature extraction under diverse polarization modes for refined oil spill identification and classification.

## Figures and Tables

**Table 1:** Description of SAR images and oil spill scenarios used in this study.

No.	Test Site Region	Data Source	Acquisition Time (UTC)	Slick Information
1	Norway, North Sea	Radarsat-2/C-band	8 June 2011, 17:27	Different oil types
2	Gulf of Mexico	Radarsat-2/C-band	8 May 2010, 12:01	Natural crude oil seeps
3	Gulf of Mexico	Radarsat-2/C-band	8 May 2015, 23:53	Nearshore oil spill
4	Philippines	ALOS/L-band	27 August 2006, 14:22	Heavy oil spill from a tanker
5	Suez Canal	Sentinel-1A	26 April 2015	illegal discharge from ships

**Table 2:** Comparison of DP scattering vectors and their FP covariance matrix relationships.

Dual-Polarimetric Structure	Scattering Vector	Corresponding Relationship to Full-Polarimetric Covariance Matrix
Cloude [17]	$k_{C}={\left[\begin{array}{cc} S_{VV} & S_{VH} \end{array}\right]}^{T}$	${\langle C\rangle }_{C}=\left[\begin{array}{cc} \langle {\left|S_{VV}\right|}^{2}\rangle & \langle S_{VV}S_{VH}^{*}\rangle \\ \langle S_{VH}S_{VV}^{*}\rangle & \langle {\left|S_{VH}\right|}^{2}\rangle \end{array}\right]=\left[\begin{array}{cc} C_{33} & C_{23}^{*}/\sqrt{2}\\ C_{23}/\sqrt{2} & C_{22}/2 \end{array}\right]$
Ji and Wu [20]	$k_{J}={\left[\begin{array}{cc} S_{VV} & 2S_{VH} \end{array}\right]}^{T}$	${\langle C\rangle }_{J}=\left[\begin{array}{cc} \langle {\left|S_{VV}\right|}^{2}\rangle & 2\langle S_{VV}S_{VV}^{*}\rangle \\ 2\langle S_{VH}S_{VV}^{*}\rangle & 4\langle {\left|S_{VH}\right|}^{2}\rangle \end{array}\right]=\left[\begin{array}{cc} C_{33} & \sqrt{2}C_{23}^{*}\\ \sqrt{2}C_{23} & 2C_{22} \end{array}\right]$
Liang [16]	$k_{L}={\left[\begin{array}{cc} S_{VV} & \sqrt{2}S_{VH} \end{array}\right]}^{T}$	${\langle C\rangle }_{L}=\left[\begin{array}{cc} \langle {\left|S_{VV}\right|}^{2}\rangle & \sqrt{2}\langle S_{VV}S_{VH}^{*}\rangle \\ \sqrt{2}\langle S_{VH}S_{VV}^{*}\rangle & 2\langle {\left|S_{VH}\right|}^{2}\rangle \end{array}\right]=\left[\begin{array}{cc} C_{33} & C_{23}^{*}\\ C_{23} & C_{22} \end{array}\right]$

**Table 3:** Statistical characteristics (mean/standard deviation) of different entropy types in representative sample areas across various oil spills scenarios.

	Measurement Index	Mean/Standard Deviation			
**Data Scenario/Class Label**		*H*	*H_C_*	*H_J_*	*H_L_*
Radarsat-2: Oil Type Differences	Crude	0.7677/0.1008	0.5314/0.1213	0.8841/0.0878	**0.7303**/0.1229
	Emulsion	0.7032/0.091	0.4120/0.0962	**0.7984**/0.1046	0.6049/0.1152
	Plant	0.3997/0.093	0.1998/0.0632	0.4963/0.119	**0.3247**/0.0931
	Sea	0.237/0.058	0.1129/0.0351	0.3135/0.083	**0.1919**/0.0563
Radarsat-2: Thickness Differences	Thick	0.9328/0.0297	0.8539/0.0802	0.8785/0.0725	**0.9391**/0.0439
	Thin	0.83161/0.0844	0.6591/0.1277	0.9251/0.0591	**0.8388**/0.1041
	Sea	0.5758/0.0923	0.3737/0.0904	0.7594/0.1062	**0.5596**/0.1107
Radarsat-2: Nearshore Oil Spill	Thick	0.362/0.10044	0.1375/0.0516	**0.3675**/0.1133	0.2301/0.0801
	Thin	0.30262/0.0933	0.1153/0.0426	**0.3171**/0.0993	0.1948/0.0677
	Sea	0.14941/0.0376	0.0649/0.0211	0.1923/0.0564	**0.1132**/0.0352
	Land	0.79176/0.0884	0.6526/0.1294	0.9109/0.0804	**0.8286**/0.1127
ALOS-1: Tanker Oil Spill	Thick	0.90049/0.0493	0.6976/0.0933	0.9518/0.0406	**0.8772**/0.0859
	Thin	0.75208/0.0835	0.5528/0.0713	0.9272/0.0489	**0.7643**/0.0720
	Sea	0.22518/0.0588	0.1333/0.0456	0.3602/0.1007	**0.2248**/0.0709

**Table 4:** The MC results of different entropies in sample areas across various oil spill scenarios.

		Measurement Index	MC		
Data Scenario/Class Label			*H_C_*	*H_J_*	*H_L_*
Scene 1	Radarsat-2: Oil Type Differences	Crude	0.18188	−0.07069	**0.02495**
		Emulsion	0.26108	**−0.06339**	0.07512
		Plant	0.3334	−0.10775	**0.10359**
		Sea	0.35471	0.13862	**0.10544**
Scene 2	Radarsat-2: Relative Thickness	Thick	0.04416	0.03002	**−0.00329**
		Thin	0.11569	−0.05317	**−0.00433**
		Sea	0.21313	−0.13193	**0.01681**
Scene 3	Radarsat-2: Nearshore Oil Spill	Thick	0.46246	**0.00315**	0.23618
		Thin	0.44925	**−0.03557**	0.20901
		Sea	0.3741	−0.1364	**0.11958**
		Land	0.10773	−0.06335	**−0.01284**
Scene 4	ALOS-1: Tanker Oil Spill	Thick	0.12736	−0.02775	**0.01308**
		Thin	0.15265	−0.10429	**−0.00809**
		Sea	0.25619	−0.23068	**0.00249**

**Table 5:** Comparison results of the overlap ratio of feature parameters under different DP modes across various oil spill scenarios.

		C vs. S	E vs. S	P vs. S	C vs. P	E vs. P	C vs. E	Thick vs. S	Thin vs. S	Thick vs. Thin	Thin vs. S	Thick vs. Thin	Oil vs. Sea
H	C	0.13	0.3	0.695	0.13	0.3	0.695	0.0425	**0.34**	**0.31**	0.013	0.68	0.5725
	J	0.135	0.2975	0.6975	0.135	0.2975	0.6975	0.4225	0.4225	0.915	0.02	0.78	0.5425
	L	**0.13**	**0.2875**	**0.6925**	**0.13**	**0.2875**	**0.6925**	0.06	0.3525	0.36	**0.01**	**0.6533**	**0.525**
A	C	0.22	**0.265**	0.865	0.22	**0.265**	0.865	**0.035**	**0.3375**	**0.3225**	0.0167	0.6733	0.53
	J	**0.2125**	0.35	0.715	**0.2125**	0.35	0.715	0.4	0.4075	0.8975	0.02	0.76	0.565
	L	0.215	0.3425	**0.7**	0.215	0.3425	**0.7**	0.055	0.34	0.375	**0.01**	**0.67**	0.56
α	C	0.1775	0.315	0.7875	0.1775	0.315	0.7875	0.0375	0.4	0.3425	0.0167	0.6967	0.575
	J	**0.14**	**0.27**	**0.7275**	**0.14**	**0.27**	**0.7275**	**0.03**	**0.3575**	0.34	**0.0033**	**0.67**	0.5575
	L	0.1675	0.275	0.7575	0.1675	0.275	0.7575	0.0375	0.375	**0.3275**	0.0233	0.6933	**0.555**
PH	C	0.1425	**0.2925**	0.71	0.1425	**0.2925**	0.71	**0.0425**	0.34	**0.3025**	0.0233	**0.6733**	0.56
	J	0.155	0.3025	0.705	0.155	0.3025	0.705	0.3975	0.4075	0.9325	**0.0133**	0.75	0.5475
	L	**0.1275**	0.3275	**0.6775**	**0.1275**	0.3275	**0.6775**	0.055	**0.335**	0.3625	0.0167	0.68	**0.5475**
C1	C	**0.195**	0.345	**0.6825**	**0.195**	0.345	**0.6825**	0.5475	**0.395**	0.77	**0.01**	0.77	**0.5275**
	J	0.63	0.8675	0.695	0.63	0.8675	0.695	0.41	0.4175	0.8975	0.6867	0.77	0.5425
	L	0.27	**0.295**	0.8775	0.27	**0.295**	0.8775	**0.2175**	0.8525	**0.36**	0.0633	**0.67**	0.5475
C2	C	0.21	0.3475	**0.685**	0.21	0.3475	**0.685**	**0.035**	**0.3375**	**0.305**	0.0133	0.6667	**0.53**
	J	0.1533	0.33	0.69	0.1533	0.33	0.69	0.3975	0.41	0.9025	0.0167	0.77	0.5675
	L	**0.1425**	**0.2875**	0.695	**0.1425**	**0.2875**	0.695	0.055	0.35	0.3725	**0.0133**	**0.6467**	0.54
C3	C	0.2	0.3375	0.695	0.2	0.3375	0.695	**0.0425**	0.34	**0.305**	0.01	0.6767	0.5375
	J	0.15	0.3	0.705	0.15	0.3	0.705	0.4	0.4025	0.9	0.0133	0.8333	0.5575
	L	**0.13**	**0.2875**	**0.6925**	**0.13**	**0.2875**	**0.6925**	0.06	**0.3375**	0.3525	**0.01**	**0.66**	**0.53**
C4	C	**0.1975**	**0.335**	0.7	**0.1975**	**0.335**	0.7	0.76	0.48	0.535	**0.01**	0.9467	0.535
	J	0.3475	0.6075	**0.6925**	0.3475	0.6075	**0.6925**	0.4	**0.4125**	0.92	0.53	0.7667	0.5525
	L	0.48	0.3825	0.8675	0.48	0.3825	0.8675	**0.0975**	0.595	**0.3575**	0.1933	**0.66**	**0.5325**

**Table 6:** Overlap ratio results of polarimetric features sets under different DP modes with Sentinel-1A data.

	Class Label	H	Alpha	A	PH	HA	H(1-A)	A(1-H)	(1-H)(1-A)
Polarimetric Feature									
thick vs. sea	C	**0.0134**	0.025	0.01	0.04	0.495	0.05	**0.005**	0.3
	J	0.4832	**0.02**	0.61	0.635	0.6	0.615	0.595	0.6
	L	0.0201	0.025	**0.005**	**0.015**	**0.09**	**0.01**	0.03	**0.02**
thin vs. sea	C	0.1678	0.4	0.395	0.23	**0.205**	0.285	0.22	0.275
	J	0.1342	0.305	0.24	0.225	0.225	0.22	0.24	**0.225**
	L	**0.1275**	**0.3**	**0.21**	**0.22**	0.98	**0.21**	**0.21**	0.475
thick vs. thin	C	**0.0872**	**0.095**	**0.09**	0.12	0.315	**0.09**	0.095	0.145
	J	0.4832	0.1	0.535	0.525	0.51	0.535	0.54	0.525
	L	0.1074	0.1	0.1	**0.095**	**0.1**	0.1	**0.095**	**0.09**

**Table 7:** Classification accuracies of the random forest classifier with Sentinel-1A oil spill data.

Class/Accuracy	Oil	Seawater
Producer Accuracy	86.87%	99.3%
User’s Accuracy	86.34%	99.36%
Average Accuracy	92%	
Kappa	0.8594	

## Data Availability

Data available on request from the authors upon reasonable requests.
